# Anti-cancer agent 3-bromopyruvate reduces growth of MPNST and inhibits metabolic pathways in a representative in-vitro model

**DOI:** 10.1186/s12885-020-07397-w

**Published:** 2020-09-18

**Authors:** Christian Linke, Markus Wösle, Anja Harder

**Affiliations:** 1 Faculty of Health Sciences, joint Faculty of the Brandenburg University of Technology Cottbus – Senftenberg, the Brandenburg Medical School Theodor Fontane and the University of Potsdam, Potsdam, Brandenburg an der Havel Germany; 2 Clinic for Radiotherapy and Radiation Oncology, Dessau City Hospital, Dessau-Roßlau, Germany; 3 Institute of Neuropathology, University Hospital Münster, Münster, Germany; 4 Institute of Pathology, Brandenburg Medical School Theodor Fontane, Dessau City Hospital, Auenweg 38, 06847 Dessau-Roßlau, Germany

**Keywords:** MPNST, NF1, 3-BrPA, Glycolysis, Mitochondrial respiration, p53, Starvation, Cell cycle, PA28, B8 fibroblasts

## Abstract

**Background:**

Anticancer compound 3-bromopyruvate (3-BrPA) suppresses cancer cell growth via targeting glycolytic and mitochondrial metabolism. The malignant peripheral nerve sheath tumor (MPNST), a very aggressive, therapy resistant, and Neurofibromatosis type 1 associated neoplasia, shows a high metabolic activity and affected patients may therefore benefit from 3-BrPA treatment. To elucidate the specific mode of action, we used a controlled cell model overexpressing proteasome activator (PA) 28, subsequently leading to p53 inactivation and oncogenic transformation and therefore reproducing an important pathway in MPNST and overall tumor pathogenesis.

**Methods:**

Viability of MPNST cell lines S462, NSF1, and T265 in response to increasing doses (0–120 μM) of 3-BrPA was analyzed by CellTiter-Blue® assay. Additionally, we investigated viability, reactive oxygen species (ROS) production (dihydroethidium assay), nicotinamide adenine dinucleotide dehydrogenase activity (NADH-TR assay) and lactate production (lactate assay) in mouse B8 fibroblasts overexpressing PA28 in response to 3-BrPA application. For all experiments normal and nutrient deficient conditions were tested. MPNST cell lines were furthermore characterized immunohistochemically for Ki67, p53, bcl2, bcl6, cyclin D1, and p21.

**Results:**

MPNST significantly responded dose dependent to 3-BrPA application, whereby S462 cells were most responsive. Human control cells showed a reduced sensitivity. In PA28 overexpressing cancer cell model 3-BrPA application harmed mitochondrial NADH dehydrogenase activity mildly and significantly failed to inhibit lactate production. PA28 overexpression was associated with a functional glycolysis as well as a partial resistance to stress provoked by nutrient deprivation. 3-BrPA treatment was not associated with an increase of ROS. Starvation sensitized MPNST to treatment.

**Conclusions:**

Aggressive MPNST cells are sensitive to 3-BrPA therapy in-vitro with and without starvation. In a PA28 overexpression cancer cell model leading to p53 inactivation, thereby reflecting a key molecular feature in human NF1 associated MPNST, known functions of 3-BrPA to block mitochondrial activity and glycolysis were reproduced, however oncogenic cells displayed a partial resistance. To conclude, 3-BrPA was sufficient to reduce NF1 associated MPNST viability potentially due inhibition of glycolysis which should lead to the initiation of further studies and promises a potential benefit for NF1 patients.

## Background

Neurofibromatosis type 1 (NF1) associated malignant peripheral nerve sheath tumors (MPNST) still do not respond well to chemotherapy and increase mortality of NF1 patients markedly. In general, malignant tumors characteristically prefer aerobic glycolysis due to gene mutations responsible for metabolic functions. We therefore investigated the effect of the anticancer compound 3-bromopyruvate (3-BrPA), a small alkylating compound that specifically suppresses cancer cell metabolism. Due to its structural similarity to lactate and pyruvate, 3-BrPA selectively enters cancer cells through monocarboxylic acid transporters which are poorly expressed in normal cells. Intracellular 3-BrPA promotes cytotoxic effects via targeting glycolytic and mitochondrial energy metabolism [1–3].

Tumor cells prefer the utilization of adenosine triphosphate (ATP) via aerobic glycolysis which is known as the “Warburg effect” [4]. Compared to mitochondrial respiration, aerobic glycolysis offers survival advantages such as faster ATP production and increased tolerance towards fluctuations in oxygen supply [5]. In addition, the conversion of pyruvate into lactate creates an acidic cellular environment toxic to normal cells. Tumor cells sustain anabolic processes using glucose and can adapt to increased levels of reactive oxygen species (ROS) [6–8]. Overactive glycolysis additionally inhibits mitochondrial respiration since glycolytic enzymes and mitochondria compete for the cytoplasmatic pool of adenosine diphosphate (ADP). Moreover, up-regulation of hexokinase isoform II (HK-II) in cancer cells has been shown to associate with voltage-dependent anion channels (VDAC) in the outer membrane of mitochondria and thought to be highly relevant for cancer cell survival [9, 10]. VDAC with bound HK-II is involved into the regulation of cell death via release of pro-apoptotic factors into cytosol such as cytochrome c (cyt c), apoptosis inducing factor (AIF) and Bcl-2-associated X protein (Bax) [11, 12].

Our analyses investigated the in-vitro effects of 3-BrPA on NF1 associated MPNST to shine a light on its repressive metabolic capacity and cytotoxic activity. From the central nervous system (CNS) counterpart, the glioma, we have learned that glioma cells undergo a metabolic reprogramming due to isocitrate dehydrogenase mutations [13]. Such as in MPNST, CNS glioma often accumulate *TP53* mutations that are associated with disturbances in DNA repair, cell cycle arrest, deregulation of apoptosis, and other important pathways. The development of the glial, but peripheral nervous system tumor type, the MPNST, similarly involves deregulation of cell-cycle regulators such as tumor suppressors p53, cyclin D1 and others. MPNST display a high percentage of *TP53* mutations which often enhances immunohistochemical expression of p53. Mutant p53 promotes expressions of the B-cell lymphoma-extra large (Bcl-xL), an anti-apoptotic member of the Bcl-2 family, and the multifaceted oncogene, c-Myc, and contributes to cellular proliferation via gain of oncogenic activity. Since p53 mediated pathways are very important for MPNST as well as for tumor development in general, a study that investigates metabolic functions in p53 dysregulated cells bearing anti-apoptotic properties was intended. To study the specific role of 3-BrPA in detail, we therefore investigated metabolic functions in mouse fibroblasts stably expressing proteasome activator (PA) 28y (Ki antigen, REGy) encoded by proteasome activator subunit 3 (PSME3) and known to be involved in DNA damage response and cell cycle control. PA28y regulates activity, distribution, and monoubiquitylation of p53 and mediates its inactivation; thereby it contributes to oncogenic transformation [14]. Therefore, the model serves to reproduce tumor associated *TP53* inactivation under controlled cell culture conditions. Since *TP53* inactivation is present in other than glial tumors, conclusions may apply to more tumor entities and may stimulate detailed research in those. Nevertheless, we deliberately selected an invariable cell culture model that shows characteristics of MPNST cells, neglects individual additional molecular events in different human MPNST, and allows to perform reproducible and control matched cell culture experiments. Oncogenic overexpression of PA28γ represses mitochondrial cyt c release through upregulation of mitochondrial Bcl-xL levels [15].

Our investigations are intended to help understand the metabolic effects of 3-BrPA on tumor cells with a special view on MPNST patients that urgently need sufficient therapies.

## Methods

### Cell lines, cell culture, and chemicals

Human MPNST cell lines (S462, T265, and NSF1) were analyzed and have been described in detail in our previous studies [16–20]. NSF1 cells were kindly provided by Dieter Kaufmann (University Hospital Ulm, Germany). Cells were pre-cultured in Dulbecco’s modified Eagle’s medium (DMEM, ThermoFisher Scientific) supplemented with 10% heat-inactivated fetal bovine serum (FBS), 100 U/mL penicillin/streptomycin, 2 mM L-glutamine, and 1 mM sodium pyruvate. Triplicates of 8 × 10^3^ cells were seeded in 100 μL media in a 96 well format and incubated for 24 h prior to 3-BrPA (Sigma Aldrich, Merck) treatment. Then, 3-BrPA was added and cells incubated for additional 24 h. For drug treatment, phosphate buffered saline (PBS, pH 7.4) was used to dilute 3-BrPA (10 μl of 0–1200 μM stock concentration with 100 μL media per well) to a final concentration of 0–120 μM. Dose application was according to literature describing doses in a range of 10 to 5000 μM applicated to tumor cells in culture [21].

Mouse fibroblast B8 cells (kindly provided by Ralf Stohwasser, Brandenburg Technical University Cottbus-Senftenberg) which were stably transfected with plasmid pSG5 vector and encoding PSME3 cDNA harbor PA28у cDNA under a constitutive SV40 promotor were used. Those cells either displayed a three- to six-fold increased expression of PA28y (B8y) or an empty pSG5 (B8vc) vector as described previously [15, 22]. B8 fibroblasts were pre-cultured in DMEM/Ham’s F12 (1:1) medium (Biochrom, Merck) with glutamine supplemented with 10% heat inactivated FBS and G418 (250 μg/mL). Triplicates of 8 × 10^3^ cells were cultured in a 96 well format. Cell lines were cultured in an incubator with a humidified atmosphere of 5% CO_2_ at 37 °C.

### Cell viability (CTB) assay

For B8 cells, triplicates of 8 × 10^3^ cells were seeded in 100 μL media in two separate 96 well formats (two to eight independent experiments) and incubated for 24 h. Fresh medium containing either 10% or 0.2% FBS was added and cells were incubated for additional 24 h prior to 3-BrPA treatment. After 3-BrPA treatment, both B8 and MPNST cells were incubated with 20 μL of CellTiter-Blue® reagent (Promega) for 1 h and fluorescence (560 nm excitation/590 nm emission) was recorded using a plate reader and expressed as fluorescence units (FU). The background fluorescence units FU_0_ of the associated untreated unstained cells were subtracted in each measurement. Finally, we normalized the measured quantity by

1 $$ \Delta  {FU}_{norm}(c)=\frac{FU(c)-{FU}_0}{FU\left(c=0\right)-{FU}_0}, $$

whereby c is the concentration of 3-BrPA.

### DHE assays

Murine fibroblasts treated with 3-BrPA in a range of 0 to 120 μM for 24 h were stained with 10 μM dihydroethidium (DHE, Sigma-Aldrich, Merck) as an independent indicator of ROS formation. Staining was performed in triplicates at 37 °C for 45 min using 96 well microtiter plates in seven independent experiments. Stained cells were washed twice with PBS. Fluorescent cells were quantified with the plate reader at 560 nm excitation and 590 nm emission. Results are also displayed as normalized fluorescence unit differences according to Eq. (1).

### Nicotinamide adenine dinucleotide tetrazolium reductase (NADH-TR) assay

A standard NADH-TR staining protocol with minor modifications was applied to investigate the ability of mitochondrial enzyme NADH dehydrogenase (complex I) to reduce colorless nitro blue tetrazolium (NBT) chloride into dark blue formazan compound. Therefore, triplicates of living cells in a 96 well format were pre-treated with 3-BrPA and washed once with PBS to remove detached cells. Then, cells were washed once with 50 mM Tris HCl (pH 7.6), and 50 μl of staining solution was added to each well. Staining solution was prepared freshly by mixing equal amounts of solution I (50 mM Tris HCl (pH 7.6) and 0,16% NADH) and solution II (50 mM Tris HCl (pH 7.6) and 0,2% NBT) prior to application. Staining of cells was performed for 2 h at room temperature. Hereafter, the absorbance of NBT formazan deposits was measured calorimetrically at a wavelength of 620 nm using a microplate reader; the arbitrary unit of the measured quantity is the colorimetrical unit (CU). The final subtraction and normalization of the measurement results were performed according to Eq. (1). Four independent experiments were carried out.

### Lactate assay

To examine aerobic glycolysis in response to 3-BrPA, we investigated lactate uptake of B8 fibroblasts. Cellular consumption of lactate in cell culture medium is high under normal growth conditions. An increase in lactate concentration in cell culture medium thereby reflects a decrease in metabolic capacity of cells. The detection of L(+)-lactate in cell culture media was performed with Lactate Assay Kit (Sigma-Aldrich). Briefly, duplicates of B8 cell lines were seeded in 96 well plates and incubated for 24 h. Next, fresh media was added containing either 10% or 0.2% FBS and cells were incubated for additional 24 h. After incubation with 3-BrPA, 50 μl medium was removed from each well and centrifuged (13,000 g for 10 min) to remove cellular debris. To ensure that the amount of lactate in the supernatant medium was in detection range, a standard curve was assayed according to the manufacturer’s instructions. Following the manual, 5 μl medium of each sample was mixed with 2 μl probe, 2 μl enzyme mix and 41 μl lactate assay buffer (per each reaction) in a 96 well plate and incubated for 30 min at room temperature. Lactate probe fluorescence was measured at 535 nm excitation and 587 nm emission by means of a microplate reader. The results of three independent experiments are expressed as normalized FU differences according to Eq. (1).

### Protein expression of cell-cycle marker proteins

To demonstrate the expression of cell cycle and apoptosis markers, we analyzed MPNST cell lines by immunohistochemistry using commercially available antibodies against the proliferation marker protein Ki-67 (antigen Ki67, monoclonal mouse anti-human Ki67 antigen, clone MIB1, M7240, DAKO), the cyclin-dependent kinase inhibitor p21 (p21, monoclonal mouse anti-human p21^WAF1/Cip1^, M7202, DAKO), the tumor protein p53 (p53, monoclonal mouse anti-human p53 M7001, DAKO), the B-cell lymphoma 2 (bcl2, monoclonal mouse anti-human bcl2 oncoprotein, M887, DAKO), the B-cell lymphoma 6 (bcl6, monoclonal mouse anti-human bcl6 protein, M7211, DAKO), and the cyclin D1 (monoclonal rabbit anti-human cyclin D1, M3642, DAKO). Analyses were performed using standard protocols according to the manufacturer’s instructions on an autostainer (DAKO Autostainer Link 48) and human immuno-positive tumors were used as controls. Ki67 labelling was analysed conventionally as well as using analysis software pathoZoom® (smart in media, Köln, Germany).

### Statistical analyses

The U test of Mann, Whitney, and Wilcoxon was applied for univariate hypothesis testing with two independent samples in each case. A significance level of α = 0.05 was used and all confidence levels were 1 – α = 0.95 ≙ 95%. A *p* value of ≤ 0.05 was considered to be significant.

Pearson’s correlation coefficient r was used to quantify the strength of a correlation. The probabilities p of zero correlation were calculated using a one-sided association test based on Student’s t test with n - 2 degrees of freedom, where n is the sample size. The quality of estimating a correlation by a regression function was evaluated using the coefficient of determination, r^2^.

The graphical representation of the results and the statistical hypothesis testing were performed by the software package MATLAB®, version R2007a (The MathWorks, Inc., Natick, MA, USA).

## Results

### Effect of 3-BrPA on viability of human MPNST and PA28у overexpressing cells

**Fig. 1:**
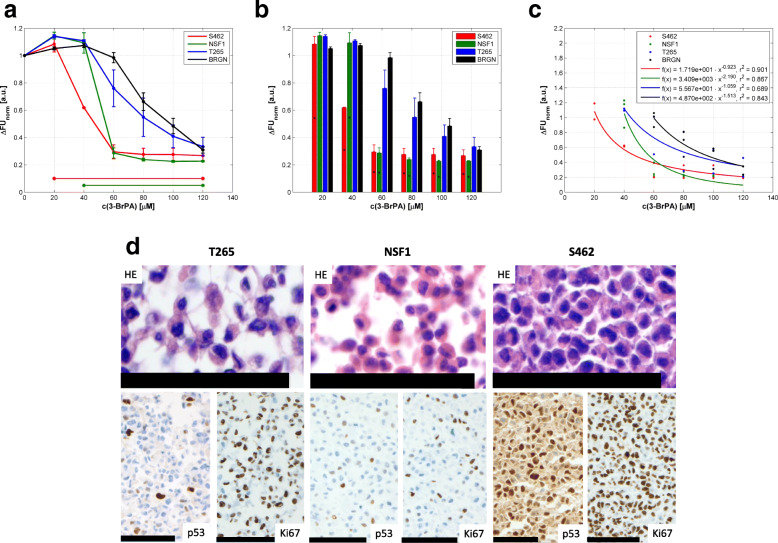
In-vitro viability under 3-BrPA treatment and expression of apoptosis and markers of cell cycle in MPNST cell lines. c(3-BrPA) – concentration of 3-BrPA; ∆FU_norm_ – normalized difference of fluorescence units. **a** Dose-dependent courses of viability in S462, NSF1, T265, and BRGN cells on agent concentration in a range of 0 to 120 μM. Mean values of normalized differences in FU according to Eq. (1)  standard errors of the mean (SEM) are presented. Concentration ranges with significant differences (*p* < 0.05) in cell viability compared to BRGN cells are marked by horizontal lines and asterisks. **b** Responses of the cell lines dependent on 3-BrPA concentration. Significant differences in cell viability compared to BRGN cells are highlighted by asterisks. **c** Correlations between cell viability and agent concentration as nonlinear regression functions. For details of statistics see Additional file 1. **d** Histopathological and immunohistochemical analysis of untreated T265 (left), NSF1 (middle) and S462 cells (right). Scale bars represent 100 μm in each photograph

Response to 3-BrPA was investigated in three human MPNST cell lines (NSF1, S462, and T265). A variable, dose-dependent reduction of cell viability was observed, and S462 cells were most responsive (Fig. 1a, b). The viabilities of S462 and NSF1 cells were significantly reduced compared to BRGN control fibroblasts: viability decreases of S462 cells were significant in an agent concentration range of 20 to 120 μM (*p* ≤ 0.031). The corresponding concentration range for the NSF1 cells was 40 to 120 μM (*p* ≤ 0.017). We found no significant decreases in the viability of T265 cells (*p* ≥ 0.605). At higher concentrations > 60 μM, control fibroblasts also showed a reduced viability. The distinct decreases in viability of the four cell lines in dependence of agent concentration are presented by means of nonlinear regression functions in Fig. 1c. Results of the correlation analyses are summarised in Additional file 1; all correlations were significant (*p* ≤ 0.005).

To demonstrate expression of marker proteins relevant for cell cycle and apoptosis in our MPNST cells, we immunohistochemically analysed Ki67, p53, bcl2, bcl6, cyclin D1, and p21. MPNST showed a moderate to high labelling for Ki67 (highest in S462 with nearly 100%) and for p53 (highest in S462 with about 80%); they are the most important criteria for usage in our study (Fig. 1d). Other markers which might be differentially regulated in MPNST were also expressed but more variable and at lower levels: Bcl6 was only expressed in NSF1 and T265, and bcl2 only weakly in S462. P21 was expressed in NSF1 and T265 at low levels. Finally, cyclin D1 was expressed in all cell lines, but only very mild in S462 and T265. Highly polymorphic MPNST cells become a small and rounded shape in culture; for details see Fig. 1d.

**Fig. 2:**
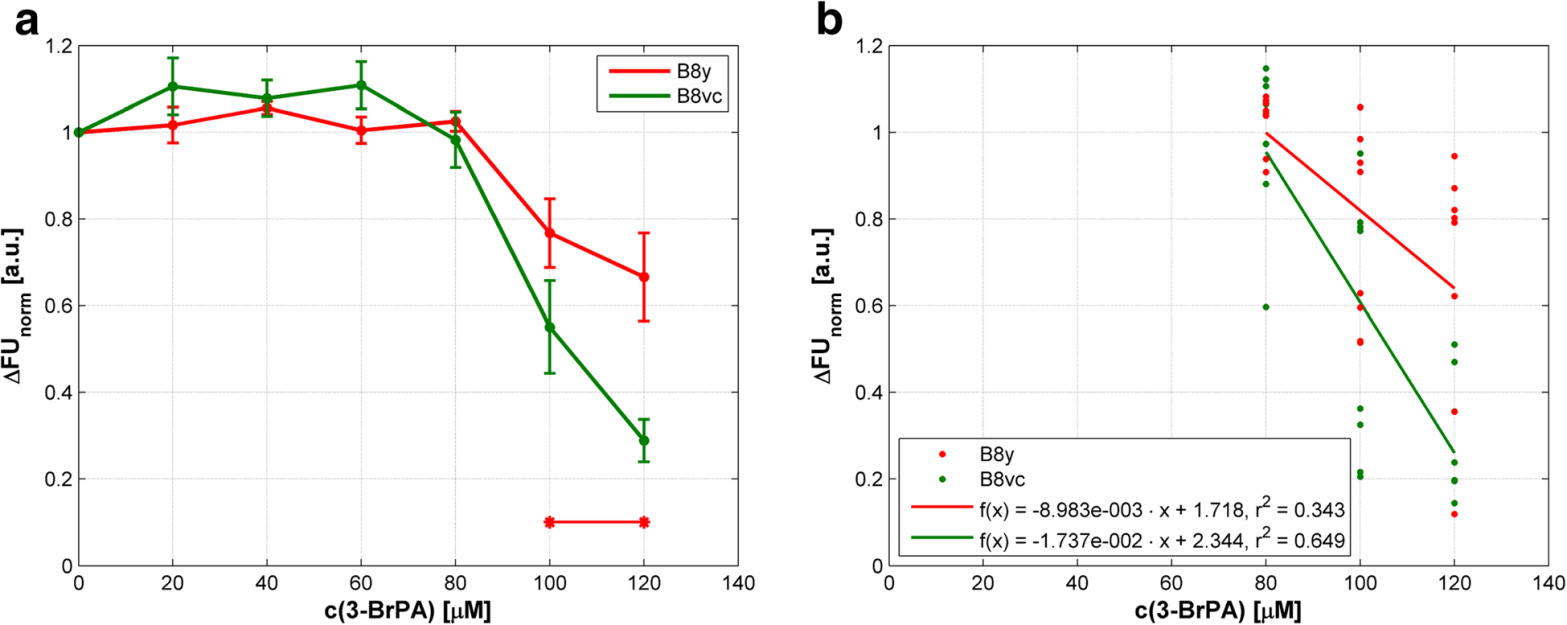
Response to 3-BrPA treatment in murine PA28y overexpressing fibroblasts (B8y) and controls (B8vc) in a cell viability assay. c(3-BrPA) – concentration of 3-BrPA; ∆FU_norm_ – normalized difference of fluorescence units. **a** Mean values of normalized differences in FU according to Eq. (1). SEM of eight independent experiments are presented. Concentration ranges with significant differences (*p* < 0.05) in cell viability compared to B8vc cells are marked by horizontal lines and asterisks. **b** Correlations between cell viability and agent concentration as regression lines. For details of statistics see Additional file 1

We additionally investigated B8 cells that should be resistant to induced apoptosis when overexpressing PA28у (B8y) due to impaired cytochrome c release into cytosol. B8у cells treated with 3-BrPA displayed a reduction of viability only at higher concentrations > 80 μM (Fig. 2a). Viability of control B8vc cells was reduced significantly and pronounced at 100 and 120 μM of 3-BrPA probably indicating higher cytotoxicity (*p* = 0.003). Figure 2a also demonstrates that PA28y overexpressing cells are partially resistant to 3-BrPA treatment. The distinct decreases in the viability of both murine cell lines on the agent concentration are presented by means of regression lines in Fig. 2b. The results of the correlation analyses are summarized in Additional file 1; all correlations were significant with *p* < 0.003.

### Effect of 3-BrPA on mitochondrial function

Such as apoptosis, p53 expression, and serum deprivation, agent 3-BrPA itself is known to cause impairment of mitochondrial functions and to increase generation of reactive oxygen species (ROS). Besides, accumulation of ROS is able to stimulate mitochondrial-dependent apoptosis. Here, we analyzed ROS generation in cells with (B8y) and without overexpression of PA28у (B8vc) under normal conditions as well as under serum deprivation (starvation).

**Fig. 3:**
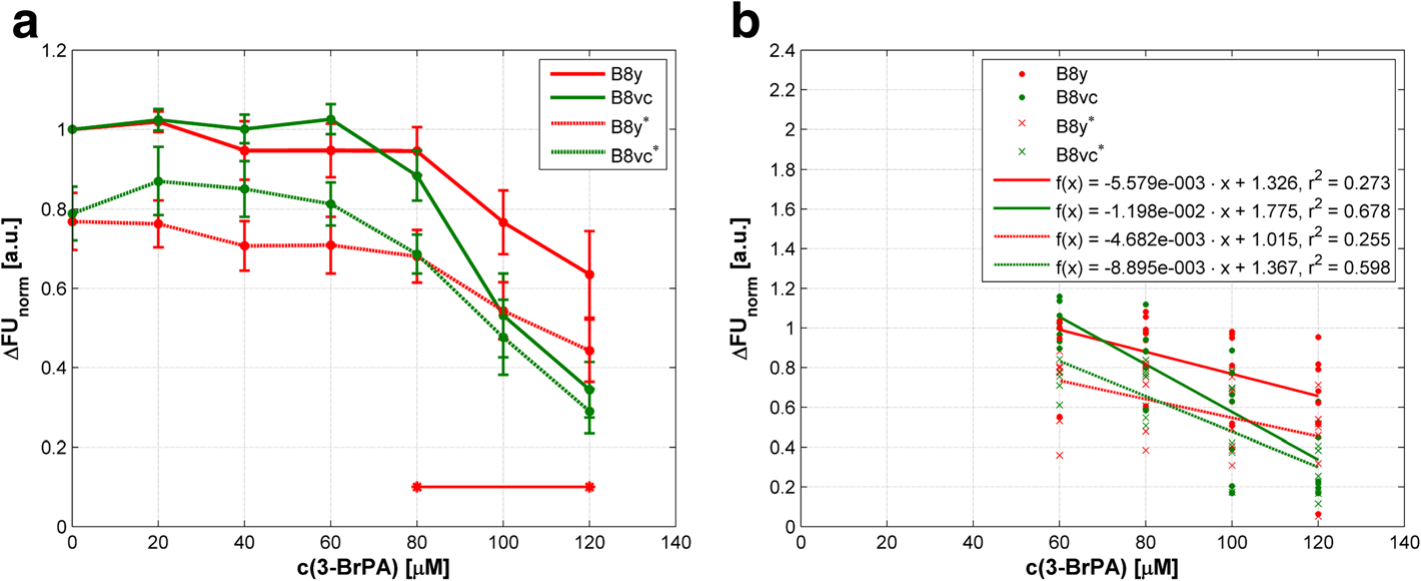
Formation of ROS in PA28y overexpressing (B8y) and control cells (B8vc) in response to 3-BrPA treatment and nutrient deprivation. c(3-BrPA) – concentration of 3-BrPA; ∆FU_norm_ – normalized difference of fluorescence units. **a** Mean values of normalized differences in FU ± SEM are presented. Results under normal conditions (10% FBS) are indicated by full lines; under serum free conditions (0.2% FBS), cell lines are marked with an asterisk and results are displayed by dotted lines. Concentration ranges with significant differences (*p* < 0.05) in ROS formation compared to starved cells are marked by horizontal lines and asterisks. **b** Correlations between ROS formation and agent concentration as regression lines. For details of statistics see Additional file 2

At higher concentrations > 60 μM 3-BrPA, a reduction of ROS was seen in B8у fibroblasts (Fig. 3a). B8vc fibroblasts which should have a controlled rate of ROS production displayed a much higher reduction of ROS levels at increasing 3-BrPA concentrations compared to PA28у overexpressing cells (Fig. 3a). The *p* values were ≤ 0.036 in the concentration range of 80 to 120 μM. The decrease of *∆FU*_*norm*_ under 3-BrPA therapy indicates a decrease of ROS production presumably due to mitochondrial complex I and III dysfunctions, and PA28у overexpression seems to temper the effect of 3-BrPA while normal fibroblasts seem to be more sensitive.

Since nutrient deprivation leads to ROS production, we analyzed if serum starvation leads to a change of ROS in 3-BrPA treated B8у cells. Under starvation, ROS levels were lower in untreated B8y and B8vc cells (Fig. 3a), nevertheless, the differences in the reduction of ROS production were not significant (*p* ≥ 0.164). But interestingly, in B8y cells ROS levels decreased at higher doses of 3-BrPA indicating the same manner of action as under normal serum conditions. Under serum free conditions and therapy with 3-BrPA, the ROS levels of the control cells closely reached levels of those cells treated under normal conditions when higher doses of 3-BrPA were applied. This indicates that nutrient deprivation induced stress affects mitochondrial functions in PA28y overexpressing and normal cells. However, in our model starvation hampers ROS production in normal cells stronger. To conclude, PA28y overexpression was associated with a mildly reduced sensitivity towards 3-BrPA treatment. Figure 3b represents the correlations of the ROS levels on the agent concentration by means of regression lines. All correlations were significant with *p* ≤ 0.006 (see Additional file 2).

**Fig. 4:**
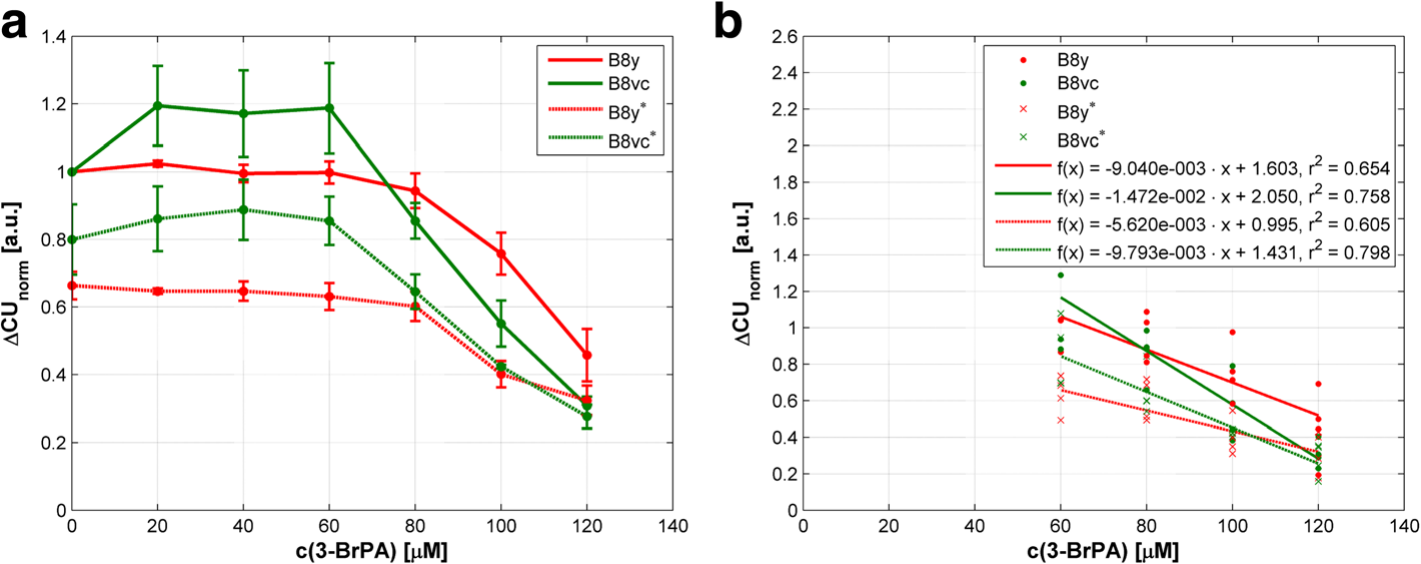
Activity of NADH dehydrogenase in PA28y overexpressing (B8y) and control cells (B8vc) after 3-BrPA treatment and nutrient deprivation. c(3-BrPA) – concentration of 3-BrPA; ∆CU_norm_ – normalized difference of colorimetric units. **a** Mean values of normalized differences in CU ± SEM are presented. Results under normal conditions (10% FBS) are indicated by full lines; under serum free conditions (0.2% FBS), cell lines are marked with an asterisk and results are displayed by dotted lines. There are no concentration ranges with significant differences (p < 0.05) in NADH dehydrogenase activity compared to starved cells. **b** Correlations between NADH dehydrogenase activity and agent concentration as regression lines. For details of statistics see Additional file 3

To address activity of mitochondrial complex I of the respiratory chain, we examined NADH dehydrogenase under 3-BrPA treatment. Comparable to the afore mentioned experiments investigating ROS generation, NADH dehydrogenase activity in untreated B8y cells was higher compared to controls. At higher doses ≥80 μM, both B8y and B8vc cells responded to 3-BrPA treatment with reduced enzyme activity, and they showed differences indicating that PA28 overexpression is associated with a higher enzyme activity and less sensitivity to treatment (Fig. 4a) although differences were not significant (*p* ≥ 0.130). Under starvation the same effect was evident, indicating a reduced mitochondrial activity (Fig. 4a), but also not significant (*p* ≥ 0.289). Figure 4b represents the correlations of the NADH dehydrogenase activity on the agent concentration by means of regression lines. All correlations were significant with *p* < 0.001 (see Additional file 3). Overall data demonstrate that 3-BrPA at least mildly harms respiratory activity of cells.

**Fig. 5:**
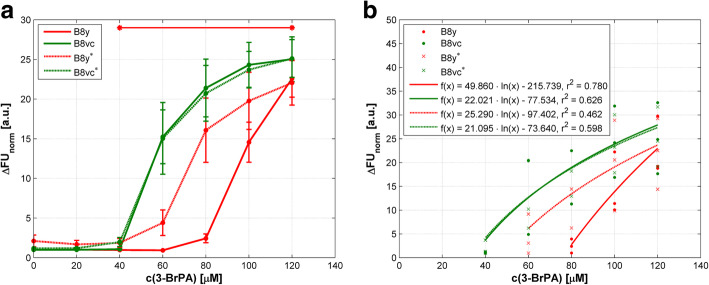
L(+)-lactate concentration was measured as indicator of L-lactate dehydrogenase activity in PA28y overexpressing (B8y) and control cells (B8vc) with respect to 3-BrPA treatment and nutrient deprivation. c(3-BrPA) – concentration of 3-BrPA; ∆FUnorm – normalized difference of fluorescence units. **a** Mean values of normalized differences in FU ± SEM are presented. Results gained with media containing 10% FBS are indicated by full lines; under starvation at 0.2% FBS, cell lines are marked with an asterisk and results are displayed by dotted lines. Concentration ranges with significant differences (*p* < 0.05) in L(+)-lactate concentration compared to starved cells are marked by horizontal lines and asterisks. **b** Correlations between L(+)-lactate concentration and agent concentration as logarithmic regression functions. For details of statistics see Additional file 4

We then analyzed L-lactate levels as an indicator for glycolytic metabolism. Under normal conditions, concentrations were not different between PA28 overexpressing and control cells (Fig. 5a). But lactate levels increased more quickly and dose-dependent due to 3-BrPA treatment in control cells compared to B8y cells: at 40 μM versus 80 μM (Fig. 5a) indicating strong inhibition of lactate consumption. The differences were significant with *p* ≤ 0.031 in concentration range of 40 to 120 μM. In control cells starvation had no influence on lactate levels indicating unchanged lactate dehydrogenase (LDH) activity. Cells overexpressing PA28 showed an earlier elevation of lactate levels when starved (Fig. 5a). The differences in LDH activity between the cell lines B8vc and B8y were not significant (*p* > 0.160). Figure 5b represents the correlations of the LDH activity on the agent concentration by means of logarithmic regression functions. All correlations were significant with *p* ≤ 0.020 (see Additional file 4).

### Effect of 3-BrPA on human MPNST under nutrient deprivation

**Fig. 6:**
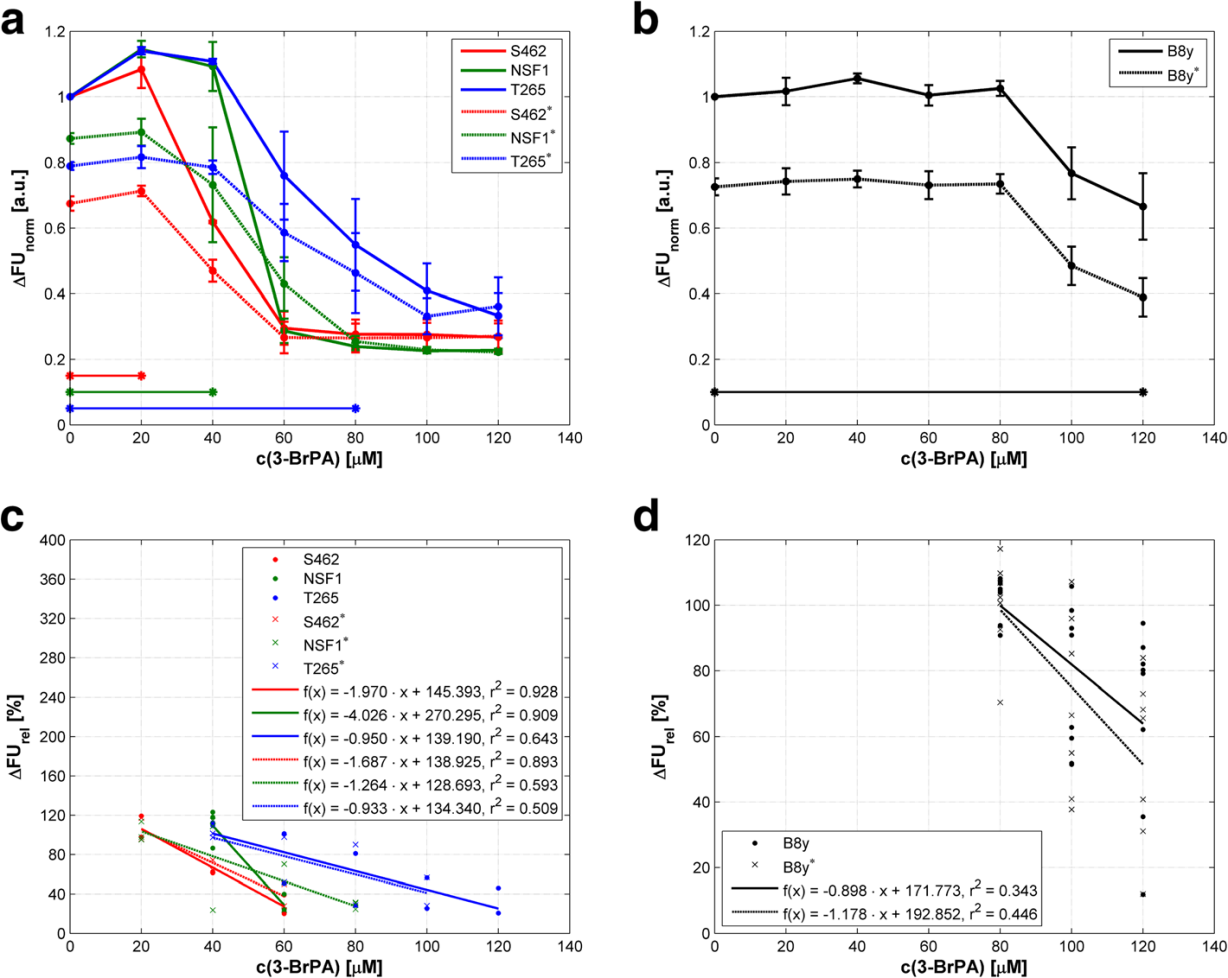
Viability of MPNST and PA28 (B8y) overexpressing cells as a function on 3-BrPA concentration without and with starvation. c(3-BrPA) – concentration of 3-BrPA; ∆FUnorm – normalized difference of fluorescence units. **a** Mean values of normalized differences in FU ± SEM are presented for MPNST cell lines. Results gained with media containing 10% FBS are indicated by full lines; under starvation at 0.2% FBS, cell lines are marked with an asterisk and results are displayed by dotted lines. Concentration ranges with significant differences (p < 0.05) in MPNST cell viability compared to starved cells are marked by horizontal lines and asterisks. **b** Mean values of normalized differences in FU ± SEM are presented for B8y cells without and with starvation. Concentration range with significant differences in B8y cell viability compared to starved cells is marked by horizontal lines and asterisks. **c** Relative changes in cell viability on 3-BrPA concentration for MPNST cell lines without and with starvation. Statistical significance (*p*-value < 0.05) is highlighted by asterisk. For details of statistics see Additional file 5.d) Relative changes in cell viability on 3-BrPA concentration for B8y cells without and with starvation. For details of statistics see Additional file 5

MPNST cell lines were investigated for their response to 3-BrPA in dependence of nutrient deprivation. Starved MPNST cells showed noticeably reduced viabilities at 0 μM of 3-BrPA that were not significant with *p* ≥ 0.100. The same effect was apparent in PA28y overexpressing and normal B8 cells (Fig. 6), but significant with *p* < 0.001. In sum, these data suggest an inhibitory effect of 3-BrPA treatment specifically on glycolytic cancer cell metabolism and therefore viability and further demonstrates the potency of our cell culture experiments.

In MPNST cells under treatment with 3-BrPA, the significant effect of starvation ebbed away with increasing concentrations. Thus, dose response to 3-BrPA treatment is clearly enhanced under starvation (Fig. 6b), but is pronounced at lower doses with significant differences in a range of 20 to 80 μM: 0 to 20 μM in S462 cells (*p* = 0.029), 0 to 40 μM in NSF1 cells (*p* ≤ 0.015), and 0 to 80 μM in T265 cells (*p* ≤ 0.029). Interestingly, although PA28y resistant cells show a reduced viability due to starvation, 3-BrPA treatment had little effect on the gradients of the curves on the agent concentration (Fig. 6b). These data indicate that PA28y overexpressing cells do not much depend on nutrient deprivation at any concentration in-vitro – as MPNST cells do. Figure 6c and d represent the relative changes in cell viability on the agent concentration by means of regression lines. The slopes of the regression lines for the cell line NSF1 without and NSF1* with starvation were quite different: − 40.3%/10 μM versus − 12.6%/10 μM (Fig. 6c). For the other cell lines, the absolute values of the differences were ≤ 2.8.%/10 μM (Fig. 6c and d). The results of the correlation analyses are summarized in Additional file 5; all correlations were significant with *p* ≤ 0.047.

## Discussion

Our experiments indicate a sensitivity of MPNST cells to treatment with 3-BrPA which can be enhanced by nutrient deprivation. In fact, MPNST show a high metabolic activity that is causal to its worse outcome. Such as elevated fluorodeoxyglucose (FDG) uptake is used for diagnosis and staging of several malignant tumors, MPNST can be differentiated from benign nerve sheath tumors via 18F-FDG uptake on positron emission tomography (18F-FDG PET) [23]. In our study we elucidated a role of 3-BrPA as a potent agent that interferes with metabolic activity in MPNST cells, although cell lines displayed a different sensitivity. The pronounced therapeutic response of MPNST paralleled by a reduced sensitivity of normal non-tumor control cells very nicely illustrates the selective impact of 3-BrPA on tumor cells. This selective sensitivity may point towards a targeted therapy approach in NF1 patients.

Serum deprivation causes stress and induces cell death. Without nutrients apoptosis, levels of ROS and caspase activity are usually elevated. Starvation is known to decrease glycolytic metabolism and to stimulate oxidative phosphorylation. In our experiments, MPNST showed dependence on glycolysis as tumor cells generally prefer the use of glycolysis (Warburg effect) and therefore reacting with a decrease of viability under starvation. Under nutrient deprivation, the therapeutic effect of 3-BrPA was enhanced. Nevertheless, at higher 3-BrPA concentrations the effect on viability was so strong that starvation became unimportant. Even so, we believe that it would be very senseful to initiate studies investigating a combination of 3-BrPA application with those substances that reduce nutrient supply. Those strategies may involve agents that prevent tumor vascularization or block recruitment of vessels, drugs that decrease blood glucose, or novel therapeutic approaches to produce a low-nutrient environment [24].

Concerning the mode of action we used murine cells that were overexpressing the proteasome activator PA28y [22] and subsequently display inactivation of p53 mediated functions. PA28y overexpression has been reported for multiple cancer entities and serves as a representative model especially for MPNST. We hypothesized that PA28y and p53 inactivation prevents cell death as it was shown previously for UV-C treatment [15]. Following the hypothesis that 3-BrPA induces apoptosis, PA28у overexpressing cells should be resistant to treatment. In fact, we were able to demonstrate that PA28y overexpressing cells responded to treatment, but not very well and only at higher concentrations of 3-BrPA. Compared to MPNST, the therapeutic response shifted to higher 3-BrPA concentrations indicating a partial resistance to treatment. This behavior favors our hypothesis that the effect of 3-BrPA is reduced when apoptosis is blocked, and inversely indicates that 3-BrPA induces apoptosis. Nevertheless, in MPNST, which show additional genetic abnormalities compared to our PA28y model, mechanisms other than p53 inactivation and apoptosis seem to allow a response to 3-BrPA.

It has been reported that 3-BrPA causes impairment of mitochondrial functions, increased ROS production and subsequent loss of cell viability in tumor cells that possess adaptation to increased ROS levels [25, 26]. Interestingly, in our experiments, we did not see an increase of ROS production due to 3-BrPA. In contrast, a decrease of ROS production was seen under treatment which approximately resembled response of cellular viability, however PA28y overexpression led to extenuation. Therefore, we assume that functions of the mitochondrial complex I and III are affected by 3-BrPA application, but in clear contrast to literature, ROS production is not enhanced due to treatment in the cell model with p53 alteration [25]. We admit that we did not clarified the origin of ROS by additional experiments since DHE assays detect both cytosolic and mitochondrial super-oxides. Nevertheless, we conclude that ROS production is not the cause of decreased cell viability in our model pointing to another mode of action.

Following our hypothesis that PA28у overexpression interferes with metabolism, we expected ROS production in response to serum starvation. Nutrient deprivation induces ROS generation that is one factor leading to cell death. PA28y overexpressing cells are resistant to apoptosis and display increased anti-apoptotic Bcl-xL levels important for homeostasis of mitochondria [27]. In our hands, ROS production was lower under nutrient deprivation, but did not differ between untreated PA28y overexpressing and control cells. We postulate that processes of ROS generation via mitochondrial complexes I and III are affected by 3-BrPA at higher concentrations, although PA28y overexpression leads to reduced sensitivity. Interestingly, we observed an effect of 3-BrPA treatment on glycolytic metabolism of B8y cells but not control cells in response to starvation. In contrast, 3-BrPA treatment did not affect LDH activity in B8y cells under normal conditions. Therefore, we conclude that starved MPNST cells are more sensitive to inhibition of glycolytic cancer cell metabolism, although we cannot rule out that an exacerbated cytotoxicity under starvation involves other cellular mechanisms as observed in B8 cells. The result also supports a selective inhibition of glycolytic metabolism in cancer cells due to 3-BrPA treatment in general as it was suggested previously. Nevertheless, our model system demonstrates that mitochondrial respiration seemed to be less affected even under starvation and potentially provides a versatile tool for cancer cells to maintain cell viability. To sum up, the effect of 3-BrPA treatment on the decrease of ROS is clearly pronounced under starvation and indicates that nutrient deprivation markedly reduces the overall viability. In control cells, the effect of starvation became unimportant at higher concentrations resembling the curve of viability of MPNST and demonstrating a difference to PA28y overexpressing cells. Especially in our model system, in PA28y overexpressing cells, ROS production is not additionally influenced by starvation and demonstrates resistance of those cells against stress. This may partially hold true for p53 altered MPNST, although other mechanisms than those mediated by PA28y and p53 overexpression seem to be additionally targeted by 3-BrPA.

Since 3-BrPA was reported to suppress energy production, we investigated NADH dehydrogenase activity associated to the mitochondrial complex I. NADH dehydrogenase converts NAD from its reduced form (NADH) to its oxidized form (NAD^+^). A higher NADH dehydrogenase activity was seen in PA28у cells under normal conditions indicating an increased rate of oxidative phosphorylation. Additionally, nutrient deprivation reduced NADH dehydrogenase activity of PA28у cells to levels of the control cell line. Since data were not significant, we herein postulate that 3-BrPA affects mitochondrial energy production only mildly. That is why starvation enhances the cytotoxic effect not impressively. Thus, 3-BrPA harms energy metabolism via respiratory activity of cells, but it is not the major mode of action in our model.

As a result of increased glycolysis in cancer cells, high amounts of pyruvate are converted to lactate instead of being directed to the mitochondrial complex I (Warburg effect). Increased lactate production is thought to be fundamental for cancer cell growth and survival, and enhanced expression of tumor-specific L-lactate dehydrogenase has been reported [28]. LDH catalyzes the conversion of pyruvate into lactate and back with concomitant interconversion of NADH and NAD^+^. The regeneration of NAD+ to NADH allows to sustain glycolytic flux in cancer cells and is thought to avoid the activity of the mitochondrial complex I and an increase in ROS production [29]. In our experiments under normal conditions, cells overexpressing PA28 showed a milder increase of supernatant levels of L-lactate in response to low doses of 3-BrPA (0–40 μM 3-BrPA). This observation was in line with our toxicity analyses and demonstrates a functional glycolytic metabolism of PA28у cells. In contrast, nutrient deprivation strongly sensitized PA28у cells to 3-BrPA treatment and inhibited lactate metabolism at already lower doses. These results suggest a potential role of PA28y in the regulation of lactate metabolism via regulation of LDH activity and with impact on 3-BrPA treatment that can be partially reversed by nutrient deprivation. The data point to an important role of lactate in tumor microenvironment, the interactive crosstalk of tumor and stromal cells, as well as biological tumor behavior and progression [30].

In summary, we demonstrated that combined inhibition of respiratory and glycolytic metabolism by 3-BrPA together with nutrient deprivation is a promising therapeutic approach.

## Conclusions

MPNST in-vitro respond dose-dependent to 3-BrPA treatment compared to control cells that showed a reduced sensitivity. In a PA28 overexpression model system leading to p53 inactivation, thereby reflecting a key molecular feature in cancer but especially in human NF1 associated MPNST, 3-BrPA application mildly harmed mitochondrial NADH dehydrogenase activity and lactate metabolism. PA28 overexpression was associated with a higher mitochondrial activity, a functional glycolysis, as well as a partial resistance to stress provoked by nutrient deprivation indicating its oncogenic potential. Interestingly, 3-BrPA treatment was not associated with an increase of ROS. In general, starvation sensitizes cells to treatment.

## Supplementary information

**Additional file 1.** Correlations between the viability of cell lines and concentration of 3-BrPA.

**Additional file 2.** Correlations between ROS level of murine cell lines and concentration 3-BrPA without and with starvation.

**Additional file 3.** Correlations between NADH dehydrogenase activity of murine cell lines and concentration of 3-BrPA without and with starvation.

**Additional file 4.** Correlations between LDH activity of murine cell lines and concentration of 3-BrPA without and with starvation.

**Additional file 5.** Correlations between relative viability of cell lines and concentration of 3-BrPA without and with starvation.

## Data Availability

The datasets used and/or analyzed during the current study are available from the corresponding author on reasonable request.

## Abbreviations

ATP: Adenosine triphosphate
ADP: Adenosine diphosphate
Bax: Bcl-2-associated X protein
Bcl2: B-cell lymphoma 2
Bcl6: B-cell lymphoma 6
Bcl-xL: B-cell lymphoma-extra large
3-BrPA: 3-bromopyruvate
CNS: Central nervous system
CTB: CellTiter-Blue®
CU: Colorimetric units
Cyt C: Cytochrome C
DHE: Dihydroethidium
DMEM: Dulbecco’s modified Eagle’s medium
DNA: Desoxyribonucleic acid
FBS: Fetal bovine serum
FDG: Fluorodeoxyglucose
FU: Fluorescence units
HCl: Hydrochloricacid
HK-II: Hexokinase isoform II
Ki: Kiel
LDH: Lactate dehydrogenase
MPNST: Malignant peripheral nerve sheath tumor
NAD: Nicotinamide adenine dinucleotide
NADH-TR: Nicotinamide adenine dinucleotide dehydrogenase tetrazolium reductase
NBT: Nitro blue tetrazolium
NF1: Neurofibromatosis type 1
PA28: Proteasome activator 28
PBS: Phosphate buffered saline
PET: Positron emission tomography
PSME3: Proteasome activator subunit 3
ROS: Reactive oxygen species
SV40: Simian vacuolating virus 40
TP53: Tumor protein 53
VDAC: Voltage-dependent anion channels
