# Supplementary material for: Anti-cancer agent 3-bromopyruvate reduces growth of MPNST and inhibits metabolic pathways in a representative in-vitro model
Source: BMC Cancer. 2020 Sep 18;20:896. doi: 10.1186/s12885-020-07397-w (PMC7501688; doi:10.1186/s12885-020-07397-w)
Supplement: Supplementary file 1 — Additional file 1. Correlations between the viability of cell lines and concentration of 3-BrPA. [file 12885_2020_7397_MOESM1_ESM.pdf]

Additional file 1

Correlations between the viability of cell lines and concentration of 3-BrPA.

Cell line	S462	NSF1	T265	BRGN	B8y	B8vc
r [1]	-0.800	-0.725	-0.802	-0.911	-0.585	-0.806
p [1]	1.778×10^{-3}	2.237×10^{-3}	5.256×10^{-3}	3.754×10^{-5}	2.661×10^{-3}	2.010×10^{-6}

r - Pearson's correlation coefficient; p - probability of zero correlation
