# Supplementary material for: Anti-cancer agent 3-bromopyruvate reduces growth of MPNST and inhibits metabolic pathways in a representative in-vitro model
Source: BMC Cancer. 2020 Sep 18;20:896. doi: 10.1186/s12885-020-07397-w (PMC7501688; doi:10.1186/s12885-020-07397-w)
Supplement: Supplementary file 2 — Additional file 2. Correlations between ROS level of murine cell lines and concentration 3-BrPA without and with starvation. [file 12885_2020_7397_MOESM2_ESM.pdf]

Additional file 2

Correlations between ROS level of murine cell lines and concentration 3-BrPA without and with starvation.

Cell line	B8y	B8vc	B8y*	B8vc*
r [1]	-0.522	-0.824	-0.505	-0.773
p [1]	4.356×10^{-3}	7.349×10^{-8}	6.101×10^{-3}	1.404×10^{-6}

r - Pearson’s correlation coefficient; p - probability of zero correlation.
