# Supplementary material for: Anti-cancer agent 3-bromopyruvate reduces growth of MPNST and inhibits metabolic pathways in a representative in-vitro model
Source: BMC Cancer. 2020 Sep 18;20:896. doi: 10.1186/s12885-020-07397-w (PMC7501688; doi:10.1186/s12885-020-07397-w)
Supplement: Supplementary file 3 — Additional file 3. Correlations between NADH dehydrogenase activity of murine cell lines and concentration of 3-BrPA without and with starvation. [file 12885_2020_7397_MOESM3_ESM.pdf]

Additional file 3

Correlations between NADH dehydrogenase activity of murine cell lines and concentration of 3-BrPA without and with starvation.

| Cell line | B8y | B8vc | B8y* | B8vc* |
|---|---|---|---|---|
| r [1] | -0.809 | -0.871 | -0.778 | -0.893 |
| p [1] | $1.482 \times 10^{-4}$ | $1.138 \times 10^{-5}$ | $3.858 \times 10^{-4}$ | $3.197 \times 10^{-6}$ |

r - Pearson's correlation coefficient; p - probability of zero correlation.
