# Supplementary material for: Anti-cancer agent 3-bromopyruvate reduces growth of MPNST and inhibits metabolic pathways in a representative in-vitro model
Source: BMC Cancer. 2020 Sep 18;20:896. doi: 10.1186/s12885-020-07397-w (PMC7501688; doi:10.1186/s12885-020-07397-w)
Supplement: Supplementary file 4 — Additional file 4. Correlations between LDH activity of murine cell lines and concentration of 3-BrPA without and with starvation. [file 12885_2020_7397_MOESM4_ESM.pdf]

Additional file 4

Correlations between LDH activity of murine cell lines and concentration of 3-BrPA without and with starvation.

Cell line	B8y	B8vc	B8y*	B8vc*
r [1]	0.879	0.746	0.658	0.734
p [1]	1.183×10^{-3}	1.401×10^{-3}	2.012×10^{-2}	1.820×10^{-3}

r - Pearson’s correlation coefficient; p - probability of zero correlation.
