# Supplementary material for: Anti-cancer agent 3-bromopyruvate reduces growth of MPNST and inhibits metabolic pathways in a representative in-vitro model
Source: BMC Cancer. 2020 Sep 18;20:896. doi: 10.1186/s12885-020-07397-w (PMC7501688; doi:10.1186/s12885-020-07397-w)
Supplement: Supplementary file 5 — Additional file 5. Correlations between relative viability of cell lines and concentration of 3-BrPA without and with starvation. [file 12885_2020_7397_MOESM5_ESM.pdf]

Additional file 5

Correlations between relative viability of cell lines and concentration of 3-BrPA without and with starvation.

Cell line	S462	NSF1	T265	B8y
r [1]	-0.963	-0.953	-0.802	-0.585
p [1]	1.978×10^{-3}	3.239×10^{-3}	5.256×10^{-3}	2.661×10^{-3}
Cell line	S462*	NSF1*	T265*	B8y*
r [1]	-0.945	-0.770	-0.713	-0.668
p [1]	4.437×10^{-3}	3.410×10^{-3}	4.704×10^{-2}	3.625×10^{-4}

r - Pearson’s correlation coefficient; p - probability of zero correlation.
